# SARS-CoV-2 nucleocapsid protein impairs stress granule formation to promote viral replication

**DOI:** 10.1038/s41421-021-00275-0

**Published:** 2021-05-25

**Authors:** Zhou-Qin Zheng, Su-Yun Wang, Zhi-Sheng Xu, Yu-Zhi Fu, Yan-Yi Wang

**Affiliations:** 1 Key Laboratory of Special Pathogens and Biosafety, Wuhan Institute of Virology, Center for Biosafety Mega-Science, Chinese Academy of Sciences, Wuhan, Hubei China; 2 University of Chinese Academy of Sciences, Beijing, China

**Keywords:** Immunology, Innate immunity

## Abstract

The newly emerging coronavirus SARS-CoV-2 causes severe lung disease and substantial mortality. How the virus evades host defense for efficient replication is not fully understood. In this report, we found that the SARS-CoV-2 nucleocapsid protein (NP) impaired stress granule (SG) formation induced by viral RNA. SARS-CoV-2 NP associated with the protein kinase PKR after dsRNA stimulation. SARS-CoV-2 NP did not affect dsRNA-induced PKR oligomerization, but impaired dsRNA-induced PKR phosphorylation (a hallmark of its activation) as well as SG formation. SARS-CoV-2 NP also targeted the SG-nucleating protein G3BP1 and impaired G3BP1-mediated SG formation. Deficiency of PKR or G3BP1 impaired dsRNA-triggered SG formation and increased SARS-CoV-2 replication. The NP of SARS-CoV also targeted both PKR and G3BP1 to impair dsRNA-induced SG formation, whereas the NP of MERS-CoV targeted PKR, but not G3BP1 for the impairment. Our findings suggest that SARS-CoV-2 NP promotes viral replication by impairing formation of antiviral SGs, and reveal a conserved mechanism on evasion of host antiviral responses by highly pathogenic human betacoronaviruses.

## Introduction

Coronaviruses are enveloped viruses that contain positive sense, non-segmented, single-stranded RNA genomes^1,2^. So far, seven human coronaviruses have been identified, including HCoV-229E, HCoV-NL63, HCoV-OC43, HCoV-HKU1, SARS-CoV, MERS-CoV, and SARS-CoV-2^1–4^. Recently, SARS-CoV-2 has caused a pandemic of acute respiratory syndromes called COVID-19 in humans^5–7^.

SARS-CoV-2 belongs to *Betacoronavirus*, and its genome sequence shares 79% identity with SARS-CoV and 50% with MERS-CoV^8^. The genome of SARS-CoV-2 codes for 16 nonstructural proteins (nsp1–nsp16) required for viral replication and pathogenesis, 8 auxiliary proteins (ORF3a, ORF3b, ORF6, ORF7a, ORF7b, ORF8, ORF9b, and ORF14), and 4 structural proteins (S, E, M, and N)^3,9^. Previous studies have demonstrated that SARS-CoV-2 uses the same receptor angiotensin-converting enzyme 2 (ACE2) as SARS-CoV to enter the cell via its S protein^6,10^. Recently, it has been reported that SARS-CoV-2 suppresses host immune responses at the early phase of infection, while activates a persistent inflammatory response at the late phase, resulting in cytokine storm and organ damage^11^.

Stress granules (SGs) are non-membranous electron-dense cytoplasmic structures/foci enriched with untranslated mRNAs. The formation and dissolvement of SGs are highly dynamic^12^. Formation of SGs can be induced upon cellular stress, such as nutrient deprivation, heat shock, UV radiation, arsenite treatment, and viral infection^13,14^. Upon viral infection, viral double-strand RNA (dsRNA) or 5′-triphosphate RNA, which are common intermediates of viral replication, binds to the protein kinase PKR, leading to conformational changes that release the C-terminal kinase domain from the N-terminal RNA-binding domain (RBD). The released kinase domain dimerizes or oligomerizes, resulting in autophosphorylation and activation. The activated PKR phosphorylates the eukaryotic translation initiation factor eIF2α, triggering Ras-GAP SH3 domain-binding protein (G3BP)- and T-cell-restricted intracellular antigen 1 (TIA-1)-dependent assembly of untranslational mRNA-enriched SGs^15,16^. Because phosphorylated eIF2α is hindered to form tRNA^Met^-GTP–eIF2 complex, synthesis of both cellular and viral proteins in the SGs is impaired following infection^15–17^.

It has been demonstrated that the induction of SGs after viral infection acts as a host antiviral strategy^18,19^. In addition to the blockade of viral gene expression by initiating translation arrest, SGs also sequester viral factors in the granules to inhibit their functions. In addition, linkage between SGs and the induction of type I IFNs has also been suggested. Certain viruses have evolved strategies to antagonize SG formation to promote their replication^20–23^. Although several mechanisms on evasion of host defense by SARS-CoV-2 have been reported^24^, it is unknown whether SG formation is targeted by SARS-CoV-2. In this study, we found that SARS-CoV-2 nucleocapsid protein (NP) impaired SG formation by inhibiting PKR autophosphorylation and activation, as well as by targeting the SG-nucleating component G3BP1. Deficiency of PKR or G3BP1 promoted replication of SARS-CoV-2. Moreover, the NP of SARS-CoV also inhibited both PKR and G3BP1, whereas MERS-CoV NP only targeted PKR. These findings reveal a relatively conserved mechanism of evasion of host defense by highly pathogenic human betacoronaviruses.

## Results

### SARS-CoV-2 evolves strategies to antagonize formation of SGs

**Fig. 1:**
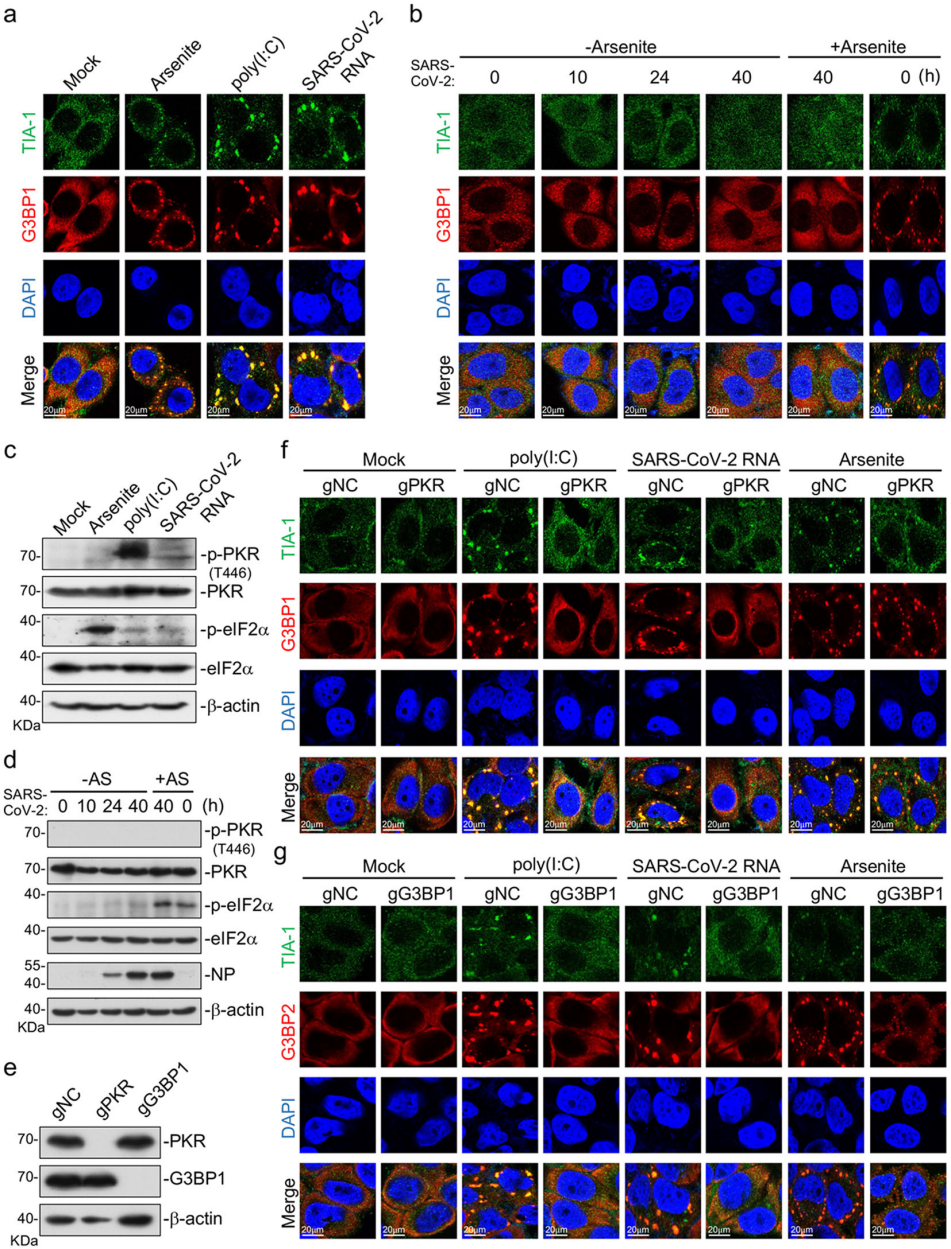
SARS-CoV-2 RNA but not SARS-CoV-2 infection induces SG formation. **a** Induction of SGs by arsenite, transfection of poly(I:C) and SARS-CoV-2 RNA. HeLa cells were treated with sodium arsenite (1 mM) for 1 h or transfected with poly(I:C) (2 μg) or SARS-CoV-2 RNA (1 μg) for 10 h. Cells were immunostained for TIA-1 (green) and G3BP1 (red). Nuclei were stained with DAPI (blue). The cells were observed with a Nikon confocal microscope under a 60× oil objective. **b** SGs were not induced by SARS-CoV-2 infection. HeLa-ACE2 cells were infected with SARS-CoV-2 (MOI = 1) for indicated times, and then untreated or treated with sodium arsenite (1 mM) for 1 h. Cells were immunostained for TIA-1 (green) and G3BP1 (red). Nuclei were stained with DAPI (blue). The cells were observed with a Nikon confocal microscope under a 60× oil objective. **c** Transfection of poly(I:C) and SARS-CoV-2 RNA and arsenite treatment induced PKR and eIF2α phosphorylation. HeLa cells were treated with sodium arsenite (1 mM) for 1 h or transfected with poly(I:C) (3 μg) or SARS-CoV-2 RNA (2 μg) for 10 h before immunoblot analysis with the indicated antibodies. **d** SARS-CoV-2 infection did not induce PKR and eIF2α phosphorylation. HeLa-ACE2 cells were infected with SARS-CoV-2 (MOI = 1) for indicated times and then untreated or treated with sodium arsenite (1 mM) for 1 h before immunoblot analysis with the indicated antibodies. **e**–**g** Formation of SGs induced by RNAs requires PKR and G3BP1. PKR and G3BP1 in HeLa cells were knocked out with the CRISPR/Cas9 system. The knockout efficiencies were detected by immunoblot with the indicated antibodies (**e**). PKR- (gPKR) (**f**), G3BP1- (gG3BP1) (**g**), and control-knockout (gNC) HeLa cells were treated with sodium arsenite (1 mM) for 1 h or transfected with poly(I:C) (2 μg) or SARS-CoV-2 RNA (1 μg) for 10 h. The cells were then immunostained for TIA-1 (green) and G3BP1(red) or G3BP2 (red). Nuclei were stained with DAPI (blue). The cells were observed with a Nikon confocal microscope under a 60× oil objective.

Previously, it has been demonstrated that formation of SGs acts as an important strategy for the host cell to antagonize viral replication^14,18,19^. We attempted to determine whether this antiviral strategy also functions to antagonize SARS-CoV-2 replication. We firstly examined SG formation in ACE2-expressing HeLa (HeLa-ACE2) cells infected with SARS-CoV-2, transfected with SARS-CoV-2 RNA or the synthetic RNA analog poly(I:C). As shown in Fig. 1a, sodium arsenite, which induces oxidative stress^25^, triggered formation of TIA-1 and G3BP1 double-positive SGs in the cytoplasm. In these experiments, transfection of SARS-CoV-2 RNA or poly(I:C) also induced the formation of TIA-1/G3BP1-positive SGs (Fig. 1a). However, TIA-1/G3BP1-positive SGs were not observed at all examined time points post SARS-CoV-2 infection (Fig. 1b). Moreover, sodium arsenite-induced formation of SGs were blocked in SARS-CoV-2-infected cells (Fig. 1b). The simplest explanation for these results is that formation of SGs is impaired by SARS-CoV-2.

It has been demonstrated that binding of viral RNA to PKR results in its autophosphorylation and subsequent eIF2α–G3BP1-mediated formation of SGs^26,27^, whereas sodium arsenite induces eIF2α–G3BP1-mediated SG formation via another kinase HRI and thus is PKR independent. Consistently, while transfection of SARS-CoV-2 RNA, poly(I:C), and sodium arsenite treatment all induced eIF2α phosphorylation, only SARS-CoV-2 RNA- and poly(I:C)-transfection but not sodium arsenite treatment enhanced PKR phosphorylation (Fig. 1c). Notably, SARS-CoV-2 infection barely induced phosphorylation of PKR and eIF2α, and had no effects on sodium arsenite-induced phosphorylation of eIF2α (Fig. 1d). Taken together, these results suggest that SARS-CoV-2 antagonizes PKR–eIF2α-mediated SG formation.

We then examined the involvement of PKR and G3BP1 in viral RNA-induced formation of SGs. Knockout of PKR by the CRISPR/Cas9 method impaired SG formation induced by transfection of poly(I:C) or SARS-CoV-2 RNA, but not by sodium arsenite treatment (Fig. 1e, f), which was consistent with previous reports that oxidative stress induces SG formation independently of PKR. However, knockout of G3BP1 impaired SG formation induced by transfection of poly(I:C) or SARS-CoV-2 RNA, as well as by sodium arsenite treatment (Fig. 1e, g). These results suggest that PKR and G3BP1 are indispensable for SARS-CoV-2 RNA-induced SG formation.

### Inhibition of SG formation promotes SARS-CoV-2 replication

**Fig. 2:**
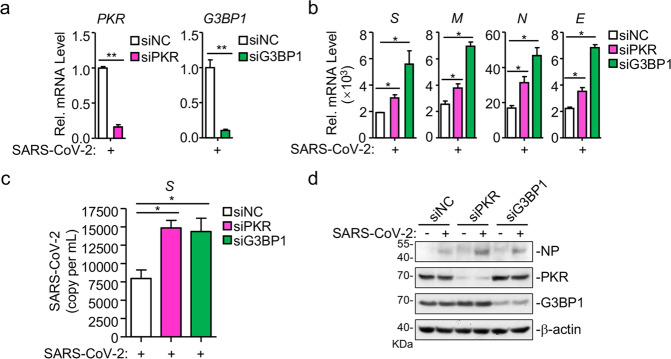
Knockdown of PKR and G3BP1 facilitates viral replication. **a**, **b** Deficiency of PKR or G3BP1 promotes the transcription of SARS-CoV-2 genes. HeLa-ACE2 cells were transfected with siRNAs specific for PKR (siPKR) or G3BP1 (siG3BP1) or control siRNA (siNC) for 48 h. The cells were then infected with SARS-CoV-2 (MOI = 0.01) for 24 h before qPCR analysis with the indicated primers. **c**, **d** Knockdown of PKR or G3BP1 promotes SARS-CoV-2 replication. HeLa-ACE2 cells were transfected with siRNAs specific for PKR (siPKR) or G3BP1 (siG3BP1) or control siRNA (siNC) for 48 h. The cells were then infected with SARS-CoV-2 (MOI = 0.01) for 24 h. Virus yield in the infected cell supernatants was quantified by qPCR analysis with primers based on RBD of SARS-CoV-2 *S* gene (**c**), and NP expression in infected cells was analyzed by immunoblot with the indicated antibodies (**d**). Graphs show means ± SD, *n* = 3. *P < 0.05, **P < 0.01 (Student’s *t-*test).

SG formation is a cellular stress response to certain RNA viruses, such as hepatitis C virus (HCV) and ZIKV, resulting in the inhibition of viral replication^20,28,29^. We next examined the roles of SGs in SARS-CoV-2 replication. We found that knockdown of PKR or G3BP1 significantly enhanced replication of SARS-CoV-2 genome in HeLa-ACE2 cells (Fig. 2a, b). Furthermore, production of progeny virus in PKR- or G3BP1-knockdown cells was significantly increased in comparison with the control cells (Fig. 2c). Consistently, the level of viral protein NP, which is another marker for viral replication, was also higher in PKR- and G3BP1-knockdown cells following SARS-CoV-2 infection (Fig. 2d). Taken together, these results suggest that PKR–G3BP1-mediated SG formation suppresses replication of SARS-CoV-2.

### SARS-CoV-2 NP inhibits SG formation by targeting both PKR and G3BP1

**Fig. 3:**
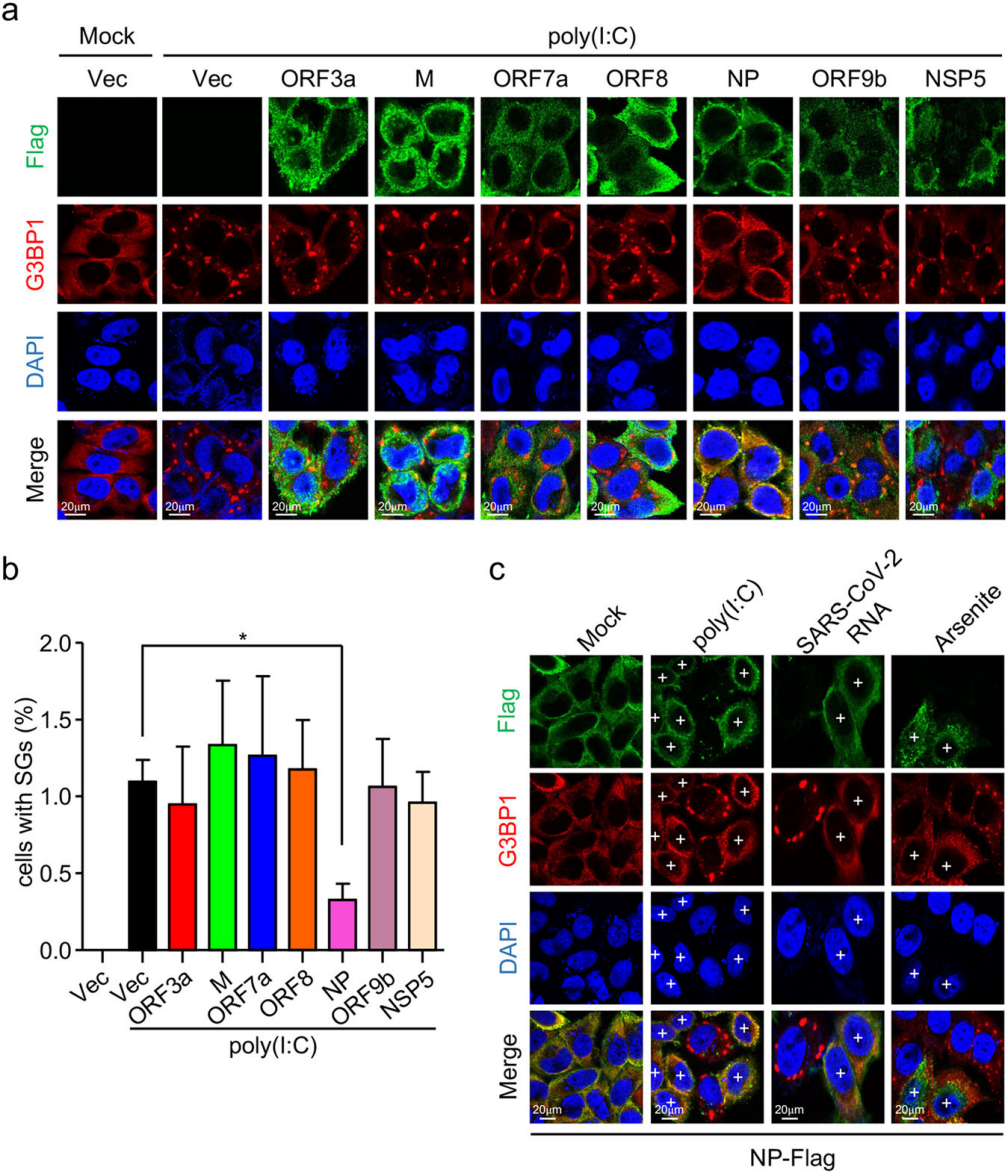
SARS-CoV-2 NP inhibits SG formation. **a** Effects of SARS-CoV-2 proteins on poly(I:C)-induced SG assembly. HeLa cells were transfected with plasmids encoding the indicated Flag-tagged SARS-CoV-2 proteins for 12 h, then transfected with poly(I:C) (2 μg) for 10 h. Cells were immunostained for Flag (green) and G3BP1 (red). Nuclei were stained with DAPI (blue). The cells were observed with a Nikon confocal microscope under a 60× oil objective. **b** Quantitative analysis of the cells (in **a**) with G3BP1 foci. The percentage of cells containing SGs was quantified and at least 100 cells were counted each time. **c** SARS-CoV-2 NP impairs poly(I:C)-, SARS-CoV-2 RNA-, and sodium arsenite-induced SG assembly. HeLa cells were transfected with SARS-CoV-2 NP-Flag plasmid for 12 h, then transfected with poly(I:C) (2 μg) or SARS-CoV-2 RNA (1 μg) for 10 h or treated with sodium arsenite (1 mM) for 1 h. Cells were immunostained for Flag (green) and G3BP1 (red). Nuclei were stained with DAPI (blue). The cells were observed with a Nikon confocal microscope under a 60× oil objective. “+” indicates the cells expressing NP. Graphs show means ± SD, *n* = 3. *P < 0.05 (Student’s *t-*test).

We next investigated the mechanisms responsible for impairment of SG formation by SARS-CoV-2. We screened for SARS-CoV-2 proteins that could inhibit poly(I:C)-induced formation of G3BP1-positive foci. The results indicated that SARS-CoV-2 NP, but not the other six examined viral proteins inhibited the formation of G3BP1-positive foci induced by transfected poly(I:C) (Fig. 3a, b). Overexpression of NP also inhibited formation of G3BP1-positive foci induced by transfection of SARS-CoV-2 RNA or sodium arsenite treatment (Fig. 3c). These results suggest that SARS-CoV-2 NP impairs viral RNA- and sodium arsenite-induced SG formation.

**Fig. 4:**
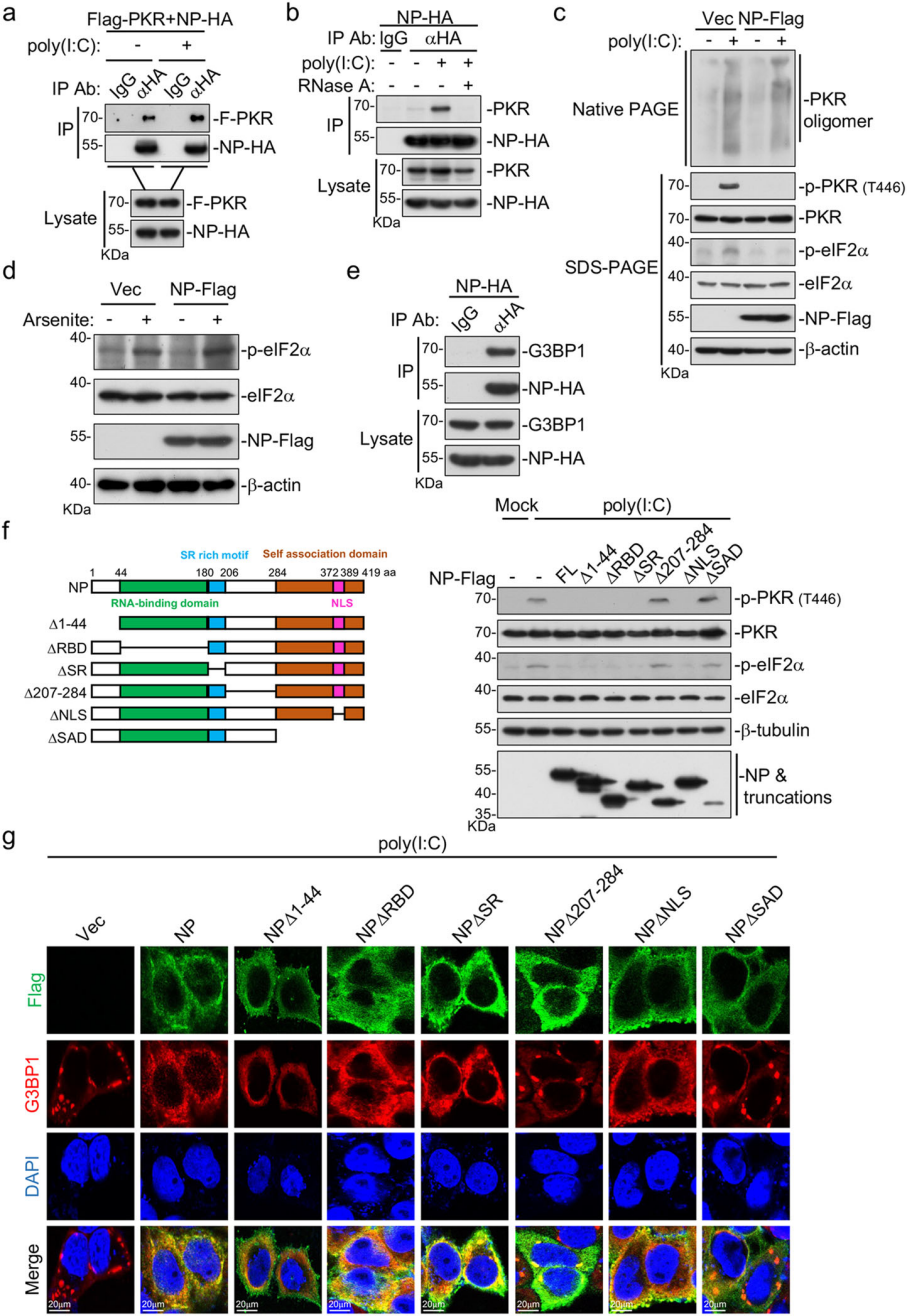
SARS-CoV-2 NP inhibits SG formation by targeting PKR and G3BP1. **a** SARS-CoV-2 NP interacts with PKR in mammalian overexpression system. HEK293T cells (5 × 10^6^) were transfected with the indicated plasmids for 24 h. The cell lysates were added with poly(I:C) (8 μg) or left untreated. Co-immunoprecipitation and immunoblot analysis were performed with the indicated antibodies. **b** Association of SARS-CoV-2 NP with endogenous PKR. HEK293T cells (5 × 10^6^) were transfected with SARS-CoV-2 NP-HA plasmid (10 μg) for 24 h. Cell lysates were left untreated or mixed with poly(I:C) (8 μg) or treated by RNase A. Co-immunoprecipitation and immunoblot analysis were performed with the indicated antibodies. **c** Effects of SARS-CoV-2 NP on poly(I:C)-induced PKR oligomerization and phosphorylation of PKR and eIF2α. HEK293T cells (5 × 10^6^) were transfected with control or SARS-CoV-2 NP-Flag plasmid (10 μg) for 24 h, and then further transfected with poly(I:C) (8 μg) for another 4 h. Cell lysates were separated by native- or SDS–PAGE as indicated and analyzed by immunoblot with the indicated antibodies. **d** Effects of SARS-CoV-2 NP on sodium arsenite-induced phosphorylation of eIF2α. HEK293T cells were transfected with control or SARS-CoV-2 NP-Flag plasmid for 20 h and then treated with sodium arsenite (1 mM) or left untreated for 1 h before immunoblot analysis with the indicated antibodies. **e** Association of SARS-CoV-2 NP with endogenous G3BP1. HEK293T cells (5 × 10^6^) were transfected with SARS-CoV-2 NP-HA plasmid (10 μg) for 20 h. Co-immunoprecipitation and immunoblot analysis were performed with the indicated antibodies. **f** Effects of NP truncations on poly(I:C)-induced phosphorylation of PKR and eIF2α. HEK293T cells were transfected with indicated plasmids for 12 h, and then transfected with poly(I:C) (2 μg) for 10 h before immunoblot analyses were performed with the indicated antibodies. **g** Effects of NP truncations on poly(I:C)-induced SG formation. HeLa cells were transfected with indicated plasmids for 12 h, then transfected with poly(I:C) (2 μg) for 10 h. Cells were immunostained for Flag-tagged NP truncations (green) and G3BP1 (red). Nuclei were stained with DAPI (blue). The cells were observed with a Nikon confocal microscope under a 60× oil objective.

Since PKR and G3BP1 play critical roles in viral RNA-induced SG assembly, we examined whether SARS-CoV-2 NP could interact with them. Co-immunoprecipitation experiments indicated that NP associated with PKR in mammalian overexpression systems (Fig. 4a). Further experiments indicated that NP associated with endogenous PKR after poly(I:C) transfection (Fig. 4b). Moreover, their association was blocked by RNase A treatment (Fig. 4b), suggesting that the interaction between NP and PKR is RNA dependent. Notably, overexpression of NP did not affect PKR oligomerization induced by transfected poly(I:C), but impaired phosphorylation of PKR and eIF2α, the hallmarks of PKR activation (Fig. 4c).

Previously, it has been shown that viral RNA induces SGs via the PKR–eIF2α–G3BP1/2 pathway, whereas sodium arsenite induces SGs via the HRI–eIF2α–G3BP1/2 axis^30^. In our study, we found that while NP inhibited poly(I:C)-triggered phosphorylation of eIF2α (Fig. 4c), it showed no effects on sodium arsenite-induced eIF2α phosphorylation (Fig. 4d), suggesting an inhibitory role of NP on PKR. Recent proteomics analysis has shown that SARS-CoV-2 NP interacts with the SG core components G3BP1 and G3BP2, as well as other RNA-binding proteins^31,32^. Co-immunoprecipitation experiments confirmed the interaction between NP and endogenous G3BP1 (Fig. 4e), which is consistent with the results that overexpression of NP, as well as SARS-CoV-2 infection also impaired arsenite-induced SG formation, which is G3BP1 but not PKR dependent (Figs. 1b and 3c). These findings indicated that NP antagonizes SARS-CoV-2 RNA-induced SG formation by targeting both PKR and G3BP1.

We next further investigated which region(s) of SARS-CoV-2 NP play key roles in the inhibition of PKR-mediated SG formation. NP consists of an N-terminal RBD (aa 45–180), a serine/arginine-rich motif (aa 176–207), a linker region (aa 208–284), and a C-terminal self-association domain (SAD, aa 285**–**419), which contains a nuclear localization sequence (aa 372–389)^33^. As shown in Fig. 4f, the C-terminus of NP that contains the linker region and the SAD was essential for the impairment of PKR and eIF2α phosphorylation induced by poly(I:C). Consistently, truncations lacking the C-terminal linker or SAD domain (NPΔ207–284 or NPΔSAD) failed to inhibit poly(I:C)-induced formation of G3BP1-positive foci, suggesting that the C-terminus of NP plays a critical role in the suppression of SG formation (Fig. 4g).

### The NPs of SARS-CoV and MERS-CoV suppress SG formation

**Fig. 5:**
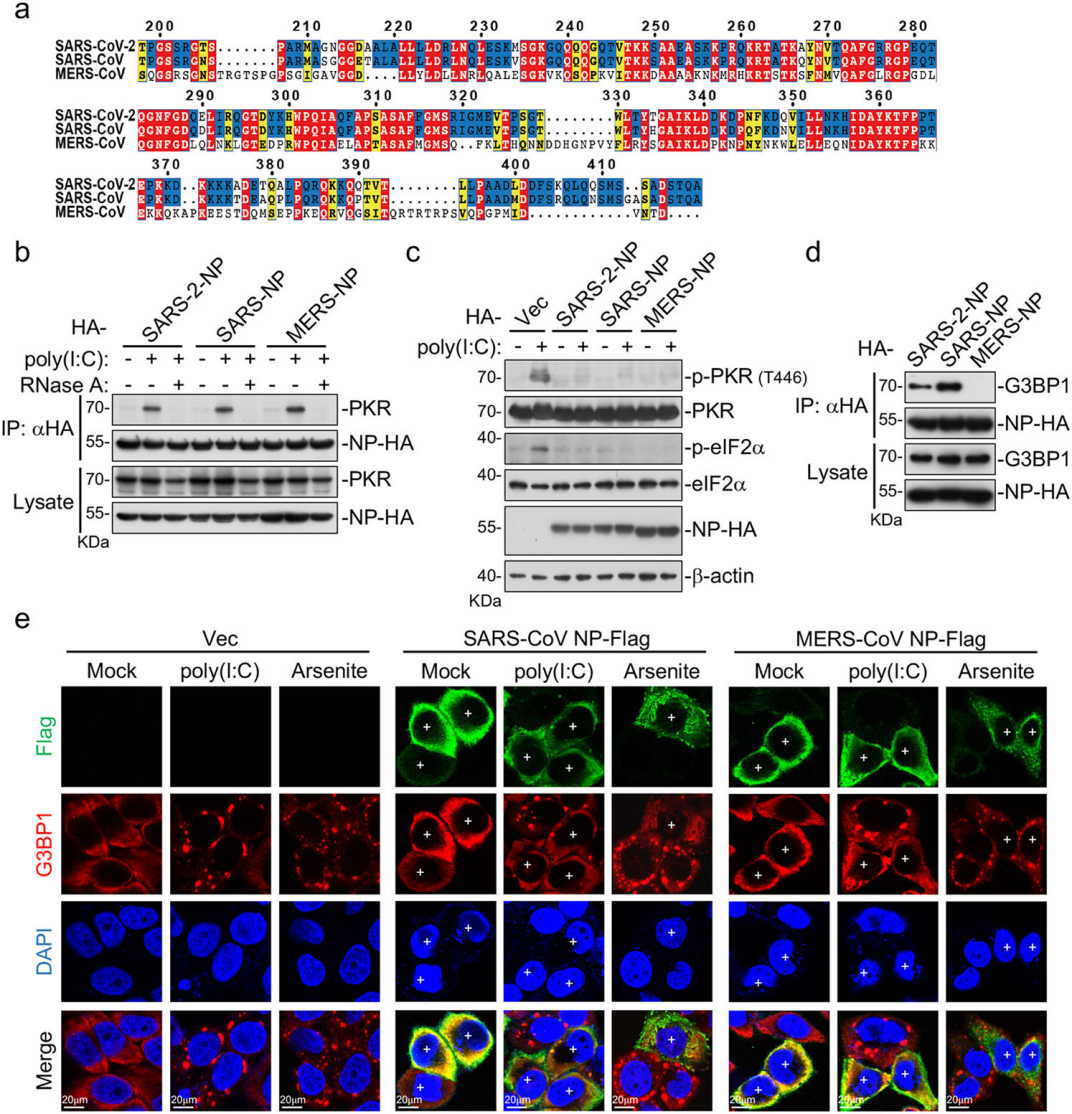
Inhibition of SG formation by NPs of three coronaviruses. **a** The amino acid sequences of NPs of SARS-CoV, SARS-CoV-2, and MERS-CoV were aligned by MAFFT (version 7.4.71) and visualized in ESPrit (version 3.0)^52^. The conserved amino acids are highlighted. Residues highlighted in red are fully conserved and those in yellow are not conserved. Residues highlighted in blue are conserved between SARS-CoV-2 and SARS-CoV, but not MERS-CoV. **b** The NPs of SARS-CoV-2, SARS-CoV, and MERS-CoV associate with endogenous PKR. HEK293T cells (5 × 10^6^) were transfected with the indicated plasmids for 24 h. The lysates were left untreated or added with poly(I:C) (8 μg) or treated by RNase A. Co-immunoprecipitation and immunoblot analysis were performed with the indicated antibodies. **c** The NPs of SARS-CoV-2, SARS-CoV, and MERS-CoV inhibit poly(I:C)-induced phosphorylation of PKR and eIF2α. HEK293T cells were transfected with the indicated plasmids for 12 h and then further transfected with poly(I:C) (2 μg) for 10 h before immunoblotting analysis with the indicated antibodies. **d** The NPs of SARS-CoV-2 and SARS-CoV, but not MERS-CoV associate with endogenous G3BP1. HEK293T cells (5 × 10^6^) were transfected with the indicated plasmids for 24 h. Co-immunoprecipitation and immunoblot analysis were performed with the indicated antibodies. **e** Effects of SARS-CoV and MERS-CoV NPs on poly(I:C)- and sodium arsenite-induced SG formation. HeLa cells were transfected with Flag-tagged SARS-CoV or MERS-CoV NP expression plasmid for 12 h, and then further transfected with poly(I:C) (2 μg) for 10 h or treated with sodium arsenite (1 mM) for 1 h. Cells were immunostained for Flag (green) and G3BP1 (red). Nuclei were stained with DAPI (blue). The cells were observed with a Nikon confocal microscope under a 60× oil objective. “+” indicates the cells expressing NPs.

**Fig. 6:**
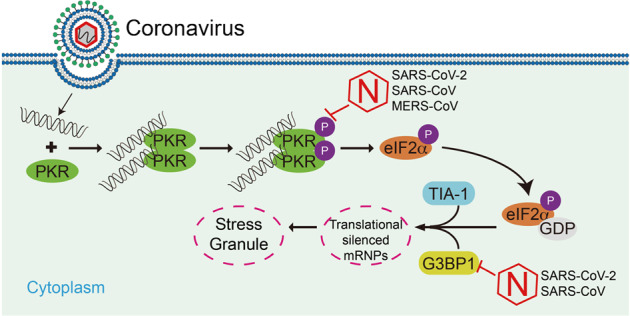
A model on inhibition of SG formation by the NPs of coronaviruses. After coronavirus infection, the viral dsRNA binds to PKR, facilitating dimerization/oligomerization and autophosphorylation of PKR. The activated PKR catalyzes the phosphorylation of eIF2α, leading to the recruitment of SG-nucleating proteins, such as TIA-1 and G3BP1, and eventually SGs assembly. The NPs of SARS-CoV-2, SARS-CoV, and MERS-CoV can inhibit PKR phosphorylation, leading to the impairment of SG formation. The NPs of SARS-CoV-2 and SARS-CoV, but not MERS-CoV can also sequester G3BP1 to impair G3BP1-mediated SG assembly.

Comparison of the C-terminus of NPs of SARS-CoV, SARS-CoV-2, and MERS-CoV showed that the amino acid sequences of NPs of SARS-CoV and SARS-CoV-2 are relatively conserved, but divergent from that of MERS-CoV (Fig. 5a). We then investigated whether the NPs of SARS-CoV and MERS-CoV play similar roles in suppression of SG formation. Similar to SARS-CoV-2 NP, the NPs of SARS-CoV and MERS-CoV interacted weakly with endogenous PKR in the absence of poly(I:C), and the interactions were enhanced following poly(I:C) stimulation and blocked by RNase A treatment (Fig. 5b). Consistently, NPs of the three coronaviruses all inhibited poly(I:C)-triggered phosphorylation of PKR and eIF2α (Fig. 5c). Interestingly, NPs of SARS-CoV, SARS-CoV-2 but not MERS-CoV interacted with G3BP1 (Fig. 5d). Further investigation revealed that NPs of SARS-CoV-2 and SARS-CoV inhibited SG formation induced by both poly(I:C) and sodium arsenite (Fig. 5e). However, MERS-CoV NP only inhibited poly(I:C)-, but not sodium arsenite-induced formation of G3BP1-positive foci in the cytoplasm (Fig. 5e). These results suggest that the NPs of SARS-CoV-2 and SARS-CoV impair formation of SGs by targeting both PKR and G3BP1, whereas MERS-CoV NP targets PKR, but not G3BP1 (Fig. 6).

## Discussion

Viral RNA-triggered, PKR–eIF2α–G3BP1-induced SGs are considered to be antiviral structures during viral infection^34^. Several mechanisms have been proposed for viral proteins to antagonize SG-mediated antiviral defense. MERS-CoV protein 4a sequesters viral RNA and prevents its binding to PKR, resulting in the inhibition of SG formation^21,22^. HCV NS5A, Japanese encephalitis virus NS2A, and Sendai virus C protein target PKR to inhibit antiviral SG formation^35–38^. Enterovirus (EV) 71 protease 3C^pro^ cleaves G3BP1 at amino acid Q326, resulting in disassembly of SGs following EV71 infection^39^. Similar mechanisms are observed for poliovirus, foot-and-mouse disease virus, feline calicivirus, and encephalomyocarditis virus^40–43^. In addition, picornavirus 2A^pro^ blocks typical SGs and induces atypical SGs via cleavage of eIF4GI to sequester cellular mRNA, but release viral mRNA^34^.

In this study, our findings suggest that SARS-CoV-2 NP impairs viral RNA-induced SG formation. Overexpression of SARS-CoV-2 NP inhibited SG formation triggered by transfected SARS-CoV-2 RNA or the RNA analog poly(I:C) (Fig. 3c). Mechanistic studies indicated that SARS-CoV-2 NP associated with the protein kinase PKR after poly(I:C) stimulation (Fig. 4a, b). It has been previously reported that PKR is activated in multiple steps^27,44^. In resting sate, PKR exists as inactive monomer and the kinase activity is autoinhibited by its N-terminal regulatory region, which includes two RBDs^27,45,46^. Upon dsRNA binding to RBDs, PKR undergoes conformational change, resulting in relief of autoinhibition and dimerization/oligomerization^27,47^. Substantially, dimerized/oligomerized PKR mediates trans-interdimer autophosphorylation at T446, which is required for its activation and recognition of the substrate eIF2α^27,44,48^. In our experiments, we found that NP interacted with PKR, inhibited autophosphorylation of PKR at T446, but not PKR dimerization/oligomerization (Fig. 4c). The simplest explanation is that the interaction of NP with PKR blocks the phosphorylation site (T446) of PKR, resulting in impaired trans-interdimer autophosphorylation. In addition to PKR, NP also interacts with the SG-nucleating protein G3BP1 (Fig. 4e) and impaired G3BP1-mediated SG formation (Fig. 3c), suggesting that SARS-CoV-2 NP targets multiple steps in SG formation. Deficiency of PKR or G3BP1 impaired poly(I:C)- or SARS-CoV-2 RNA-triggered SG formation (Fig. 1f, g), and increased SARS-CoV-2 replication (Fig. 2). These results suggest that impairment of SGs by the NP of SARS-CoV-2 represents an important mechanism for its evasion of host defense.

Domain mapping analysis of SARS-CoV-2 NP showed that its C-terminus (aa 207–419) is essential for the impairment of SG formation (Fig. 4f, g). Alignment of the C-terminus of NPs of SARS-CoV, SARS-CoV-2, and MERS-CoV revealed that SARS-CoV and SARS-CoV-2 are relatively conserved, but divergent from that of MERS-CoV. Further investigation indicated that the NP of SARS-CoV also targeted both PKR and G3BP1 to impair dsRNA-induced SG formation, whereas the NP of MERS-CoV targeted PKR, but not G3BP1 for the impairment (Fig. 5b–e). These results suggest that the roles of NPs of these coronaviruses in evasion of host defense are conserved, but not totally the same. In conclusion, our findings suggest that SARS-CoV-2 NP promotes viral replication by impairing formation of antiviral SGs, and reveal a conserved mechanism on evasion of host antiviral responses by highly pathogenic human betacoronaviruses (Fig. 6).

## Materials and methods

### Reagents, antibodies, cells, and viruses

Lipofectamine 2000 (Invitrogen); FuGene (Promega); puromycin (Thermo Fisher); SYBR Green Supermix (BIO-RAD); polybrene (Millipore); Protein G sepharose (GE Healthcare); mouse antibodies against Flag and β-actin (Sigma-Aldrich); HA (OriGene); β-tubulin and SARS-CoV-2 NP (Cell Signaling Technology); TIA-1 (Santa Cruz Biotechnology); rabbit antibodies against HA, eIF2α, and phosphor-eIF2α (Ser51) (Cell Signaling Technology); PKR and phosphor-PKR (T446); G3BP2 (Abcam); and G3BP1 (ABclonal) were purchased from the indicated companies. HEK293T, Vero E6, and HeLa cells were purchased from ATCC. HeLa-ACE2 cells (stably expressing ACE2) were constructed by lentiviral-mediated transduction. Cells were cultured in DMEM (Hyclone) supplemented with 10% fetal bovine serum (Gibco) and 1% penicillin–streptomycin (Thermo Fisher Scientific) at 37 °C with 5% CO_2_. SARS-CoV-2 (IVCAS 6.7512) was isolated from BALF collected from a patient with viral pneumonia in December of 2019 in Wuhan, China^6^. The virus was propagated in Vero E6 cells^49^. SARS-CoV-2 RNA was isolated from Vero E6 cells infected with SARS-CoV-2 for 48 h. All SARS-CoV-2-related experiments were performed in the biosafety level 3 (BSL-3) laboratory of Wuhan Institute of Virology.

### Plasmids

Mammalian expression plasmids for Flag- or HA-tagged SARS-CoV-2 NP and its truncations, SARS-CoV NP, MERS-CoV NP, and PKR were constructed by standard molecular biology techniques.

### Transfection

HeLa cells were transfected by FuGene and lipofectamine 2000. HEK293T cells were transfected by standard calcium phosphate precipitation method. Control plasmids were added to ensure that each transfection receives the same amount of total DNA.

### Stable cell lines

HEK293T cells were transfected with two packaging plasmids (pSPAX2 (7.5 μg) and pMD2.G (5 μg)) together with empty vector, or the indicated plasmids (10 μg) by calcium phosphate precipitation. Twelve hours later, the medium was replaced. Thirty-six hours later, the recombinant virus-containing medium was filtered (0.45 μm) and added to HeLa cells in the presence of polybrene (8 μg/mL). Twenty-four hours post infection, cells were selected with puromycin (0.5 μg/mL) for 7 days before experiments.

### qPCR

Total RNAs were isolated from cells and reverse-transcribed to cDNA for qPCR analysis to measure mRNA levels of the indicated genes. Data shown are the relative abundance of the indicated mRNA normalized to that of GAPDH. Primer sequences for qPCR assays were as follows:

human *GAPDH*, GAGTCAACGGATTTGGTCGT and GACAAGCTTCCCGTTCTCAG;

SARS-CoV-2 *S*, CTTCCCTCAGTCAGCACCTC and AACCAGTGTGTGCCATTTGA;

SARS-CoV-2 *M*, AATTTGCCTATGCCAACAGG and GTACGCGCAAACAGTCTGAA;

SARS-CoV-2 *E*, TCGTTTCGGAAGAGACAGGT and CACGAGAGTAAACGTAAAAAGAAGG;

SARS-CoV-2 *N*, CATTGGCATGGAAGTCACAC and

TCTGCGGTAAGGCTTGAGTT.

### Measurement of SARS-CoV-2 viral titer

Cell culture supernatant of SARS-CoV-2-infected HeLa-ACE2 cells was harvested, and viral RNA was extracted using the MiniBEST Viral RNA/DNA Extraction Kit (Takara)^6,50^. Viral RNA was eluted with RNase-free water and reverse-transcribed to cDNA for qRT-PCR. A standard curve was generated by serial dilutions (10^3^–10^9^ copies) of the plasmids encoding RBD of the SARS-CoV-2 Spike gene. The level of SARS-CoV-2 Spike gene in the cell culture supernatant was then determined by qPCR and further converted to the viral titer, as previously described^6,50^. The primers used for the SARS-CoV-2 Spike gene RBD were: 5′-CAATGGTTTAACAGGCACAGG-3′ and 5′-CTCAAGTGTCTGTGGATCACG-3′^6,50^.

### Co-immunoprecipitation and immunoblot analysis

HEK293T cells (5 × 10^6^) were lysed with 1 mL pre-lysis buffer (20 mM Tris-HCl, pH 7.4, 150 mM NaCl, 1 mM EDTA, 1% Triton X-100, 10 μg/mL aprotinin, 10 μg/mL leupeptin, and 1 mM phenylmethylsulfonyl fluoride) for 30 min on ice. Cell lysates were clarified by centrifugation at 4 °C, 12,000 r.p.m. for 15 min. For each immunoprecipitation, the lysate (400 μL) was incubated with the indicated antibodies (0.5 μg each) and protein G sepharose beads (25 μL) at 4 °C for 3–5 h. The protein-bound beads were then collected and washed three times with 1 mL of lysis buffer containing 0.5 M NaCl. Immunoblot analysis was performed by standard procedures.

### Confocal microscopy

HeLa cells were transfected with the indicated plasmids by FuGene. After transfection for 20 h, the cells were stimulated with sodium arsenite for 1 h or transfected with poly(I:C) and SARS-CoV-2 RNA by lipo2000 for 10 h. The cells were fixed with 4% paraformaldehyde for 10–15 min on ice and washed with PBS for three times, then permeabilized with 0.3% Triton X-100 on ice for 10 min and blocked in 1% BSA for 20 min at room temperature. The cells were then incubated with the indicated primary antibodies overnight at 4 °C. Alexa Fluor 488- and 555-conjugated secondary antibodies were incubated with the cells for 1 h. The nuclei were stained with DAPI for 2 min before images were acquired using Nikon confocal microscope under a 60× oil lens objective.

### PKR oligomerization assay

Analysis of PKR oligomerization was performed, as described previously^51^. HEK293T cells were lysed in 100 μL PBS containing 0.5% Triton X-100 and incubated for at least 10 min at 4 °C. Cell lysates were clarified by centrifugation at 4 °C, 10,000× *g* for 10 min. An aliquot of cell lysate (10 μL) was mixed with 5× native sample buffer (250 mM Tris-HCl, pH 6.8, 1% sodium deoxycholate, 50% glycerol, and 0.5% bromophenol blue) or 2× SDS loading buffer. The samples were analyzed by native PAGE or SDS–PAGE, respectively. The native PAGE was run at 4 °C with anode buffer (50 mM Tris-HCl, pH 9.0 and 384 mM glycine) and cathode buffer (50 mM Tris-HCl, pH 8.3, 384 mM glycine, and 1% sodium deoxycholate) at 20 mA per gel. The proteins were transferred to immobilon membrane (Millipore) by standard procedures with Towbin buffer (25 mM Tris, 192 mM glycine, 0.1% SDS, and 20% methanol) at 250 mA for 1.5 h at 4 °C. Immunoblot analysis was performed by standard procedures. The SDS–PAGE was performed by standard procedures.

### Statistics

Unpaired Student’s *t-*test was used for statistical analysis with GraphPad Prism Software; *P < 0.05 and **P < 0.01 were considered significant.
